# The differentiation of pancreatic neuroendocrine carcinoma from pancreatic ductal adenocarcinoma: the values of CT imaging features and texture analysis

**DOI:** 10.1186/s40644-018-0170-8

**Published:** 2018-10-17

**Authors:** Chuangen Guo, Xiaoling Zhuge, Qidong Wang, Wenbo Xiao, Zhonglan Wang, Zhongqiu Wang, Zhan Feng, Xiao Chen

**Affiliations:** 1 Department of Radiology, the First Affiliated Hospital, College of Medicine Zhejiang University, 79 Qingchun road, Hangzhou, 310003 China; 2 Department of Laboratory Medicine, the First Affiliated Hospital, College of Medicine Zhejiang University, 79 Qingchun road, Hangzhou, 310003 China; 3 Department of Radiology, the Affiliated Hospital of Nanjing University of Chinese Medicine, 155 Hanzhong road, Nanjing, 210029 China

**Keywords:** Pancreatic neuroendocrine carcinoma, Pancreatic ductal adenocarcinoma., Computed tomography., Texture analysis.

## Abstract

**Background:**

Imaging findings for pancreatic neuroendocrine carcinoma (PNEC) and pancreatic ductal adenocarcinoma (PDAC) often overlap. The aim of this study was to demonstrate the value of computed tomography (CT) imaging features and texture analysis to differentiate PNEC from PDAC.

**Methods:**

Twenty-eight patients with pathologically-proved PDAC and 14 patients with PNEC were included in this study. CT imaging findings, including tumor boundary, size, enhancement degree, duct dilatation and parenchymal atrophy were used to compare PDAC and PNEC. CT texture features were extracted from CT images at the arterial and portal phases.

**Results:**

More PNEC than PDAC had well-defined margins (57.1% vs 25.0%, *p* = 0.04). Parenchymal atrophy was more common in PDAC than in PNEC (67.9% vs 28.1%, *p* = 0.02). CT attenuation values (HU) and contrast ratios of PNEC inthe arterial and portal phases were higher than those of PDAC (*p* < 0.05 or 0.01). Entropy was lower and uniformity was higher in PNEC compare to PDAC at the arterial phase (p < 0.05). Contrast ratio showed the highest area under curve (AUC) for differentiating PNEC from PDAC (AUC = 0.98–0.99). Entropy and uniformity also showed an acceptable AUC (0.71–0.72).

**Conclusions:**

Our data indicate that CT imaging features, including tumor margin, enhanced degree and parenchymal atrophy, as well as texture parameters can aid in the differentiation of PNEC from PDAC.

## Background

Pancreatic neuroendocrine carcinoma (PNEC) is a rare tumor that accounts for 2–3% of pancreatic neuroendocrine neoplasms (PNENs) [1, 2]. Recently, several studies reported that PNEC usually showed hypovascular pattern in contrast-enhanced computed tomography (CT) or magnetic resonance imaging (MRI) [3–6]. In addition, ill-defined borders and lymph node invasion are also common in PNEC. These key imaging features are also critical imaging findings in pancreatic ductal adenocarcinoma (PDAC). Overlaps in imaging findings between PNEC and PDAC have been reported previously [7]. In a prior study, we found that 57% of PNEC was misdiagnosed as PDAC [6].

The treatment strategies and prognosis of PNEC and PDACs are substantially different. For PNEC, surgical therapy is available if curative resection is possible even in cases with limited metastases [8, 9]. In addition, several reports indicate that therapy with sunitinib or everolimus is also helpful for PNEC [10, 11]. Usually, the prognosis of PNEC is better than PDAC. Therefore, correctly identifying PNEC and PDAC is an important prerequistite treatment.

Previous several studies have shown that CT and MRI are useful for differential diagnosis of hypovascular pancreatic tumors [6, 12]. Recently, texture analysis that extracts, analyzes, and interprets quantitative imaging features has been widely used to diagnose, characterize and improve tumor staging and therapy response assessment in cancer field [13]. Canellas et al. [14] indicated that CT texture analysis and CT features are predictive for PNENs aggressiveness. However, to the best of our knowledge, no studies have examined differences in texture parameters between PNEC and PDAC. The aim of our study was to investigate the utility of CT imaging findings and CT texture features in identifying PNEC from PDACs.

## Material and methods

### Study population

We used medical records to identify 21 patients with surgically or biopsy-proven PNEC diagnosed between January 2012 to July 2017 accordance with the WHO 2010 classification for PNENs. Seven patients were excluded because they did not receive a preoperative CT examination or lacked dynamic contrast-enhanced CT images. We also searched the medical record from January 2017 to July 2017 and identified 78 patients with surgically or biopsy-proven PDAC. Twelve patients who did not receive CT examination or lacked dynamic contrast-enhanced CT images, while six patients whose tumor presented as dominantly cystic were also excluded. Among the remaining 60 subjects, we randomly selected 28 patients in a proportion of 1: 2 with respect to PNEC. Ultimately, a total of 28 PDAC patients and 14 PNEC patients were included in this study. Histological diagnose of PNEC were based on the following criteria: PNEC G3, > 20 mitoses per 10 HPF, Ki-67 index > 20%. This retrospective study was approved by institutional review board of the Affiliated Hospital of College of Medicine Zhejiang University and the need for formal consent of patients was waived.

### CT protocol

All CT imaging was performed using the same multidetector CT system (Brilliance 128, Philips Healthcare, Best, The Netherlands) following to a standardized protocol. Three phase images (conventional, arterial and portal venous) were obtained from each patient. The CT scanning parameters were as the following: tube voltage of 120 kV; slice thickness of 3 mm; beam collimation, 128 × 0.625 mm; and automatic tube current modulation. Contrast-enhanced CT images were obtained after intravenous administration of iohexol (300 mg/mL, Bayer Health Care Pharmaceuticals, Germany) at a rate of 3.0 mL/s via a power injector (1.5 ml/kg), followed by a 20-mL bolus of sodium chloride. The enhanced images were obtained at the arterial phase (30–35 s) and the portal phase (55–60s).

### Image analysis

The images were reviewed by two abdominal radiologists with more than six years of clinical experience. They were blind to the pathologic data. The following imaging parameters were recorded based on the unenhanced images: tumor location, size, tumor margins (well-circumscribed or ill-defined border), pancreatic duct dilatation, parenchymal atrophy (absent or present), and lymph node invasion or local invasion (confirmed by histological examination). The definition of tumor margin was obtained from a previous study [15]. Well-circumscribed was defined as smooth margins without spiculation or with less than 80% infiltration, and pancreatic duct dilation was defined as a main pancreatic duct diameter ≥ 4 mm. Quantitative data, including tumor attenuation on unenhanced CT scan, contrast ratio at arterial phase (AER) [Hounsfield Unit (HU) values of tumor/HU values of normal parenchyma measured in the arterial phase], and contrast ratio at portal venous phase (PER) (HU values of tumor/HU values of normal parenchyma measured in the portal phase), were also measured. For AER and PER measurements, the regions of interest (ROIs) were set at the solid components, avoiding necrotic or cystic components.

### Texture analysis

The images obtained at the arterial phase and portal phase were used for texture analysis. ROIs were manually drawn in every visualized tumor images in consensus by two abdominal radiologists. The necrotic components were excluded from ROIs. One of them performed the texture analysis by using Matlab2014b (MathWorks, Natick, MA, USA). The regions outside the ROI were set with the average value ofthe pixels inside the ROI in order to reduce the impact of nontarget regions. Texture data from the whole tumor was obtained. We used the filtration-histogram approach and Laplacian-of-Gaussian band-pass filters (sigma values of 0.5, 1.5 and 2.5). The texture parameters under different filters, including kurtosis, skewness, entropy and uniformity, were analyzed. The mathematical expression and means of those parameters have been described in a previous report [14].

### Statistical analysis

Data were managed and analyzed with SPSS 16.0 (SPSS Inc., Chicago, IL, USA). Quantitative data were displayed as means ± standard deviations and qualitative data were expressed as numbers (percentage). We used the χ^2^ text or Fisher exact test for categorical variables and the Mann-Whitney U test for continuous variables. Receiver operating characteristics (ROC) curve analysis was performed and the area under the curve (AUC), sensitivity, and specificity was calculated to ascertain diagnostic ability. Interobserver agreements in ROIs were assessed with Conger’s kappa test. *P* values < 0.05 were considered statistically significant.

## Result

**Fig. 1:**
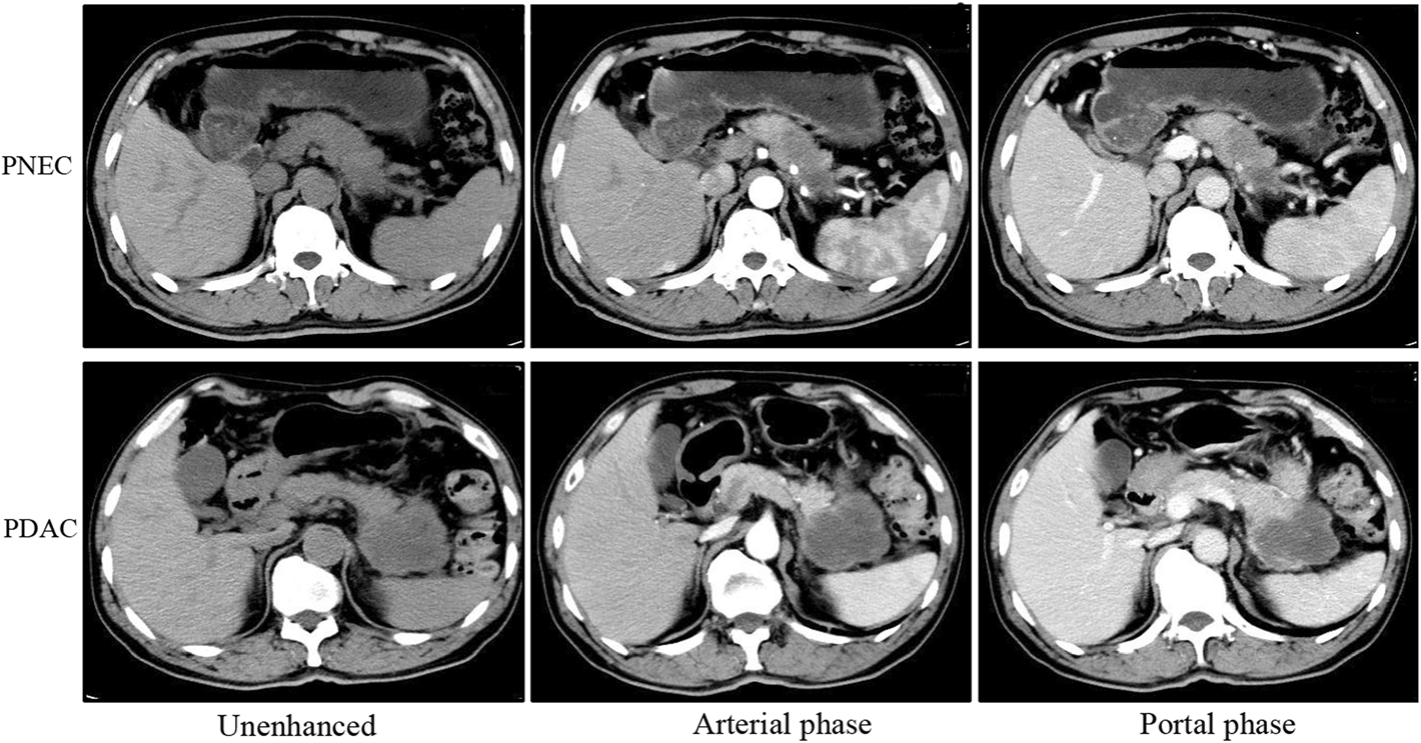
The computed tomography imaging findings in a 66-year-old woman with pancreatic neuroendocrine carcinoma (PNEC, white arrow) and a 62-year-old man with pancreatic ductal adenocarcinoma (PDAC, black arrow). Unenhanced and contrast-enhanced CT images at the arterial phase and portal phase showed ill-defined, hypovascular mass

**Fig. 2:**
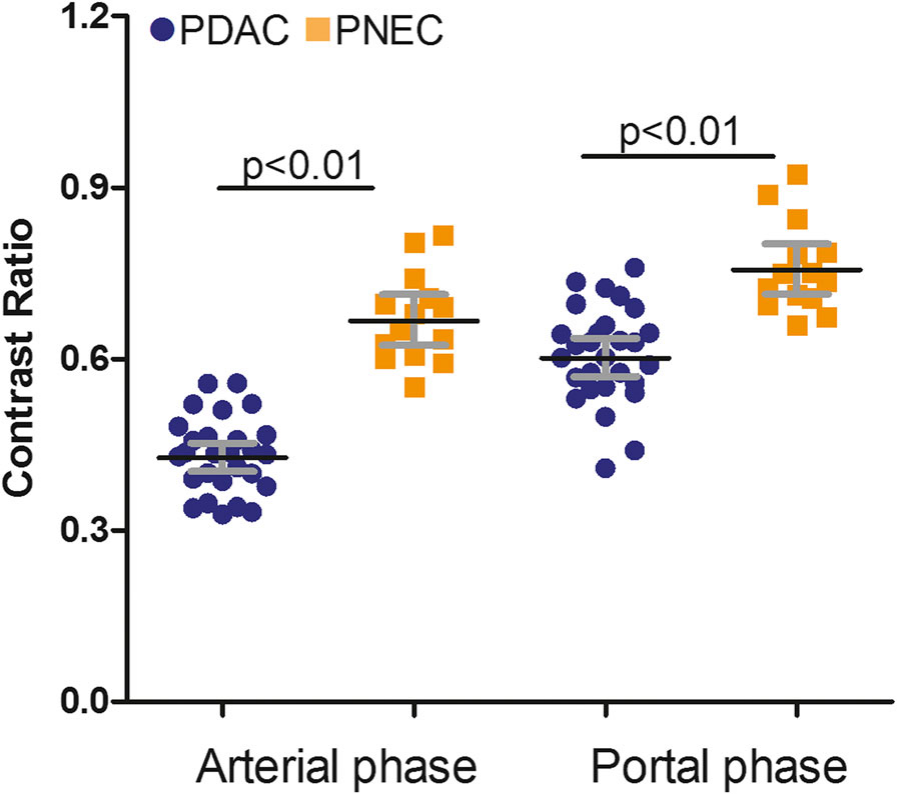
The contrast ratio in pancreatic neuroendocrine carcinoma (PNEC) and pancreatic ductal adenocarcinoma (PDAC) at the arterialand portal phases. The contrast ratios were higher in PNEC than PDAC

The characteristics of subjects are listed in Table 1. The age of patients with PNEC was lower than that of patients with PDAC(*p* < 0.05). CT images of PNEC and PDAC were provided in Fig. 1. Both two lesions showed hypovascular pattern on contrast-enhanced images. No significant differences were found in gender, size, tumor location, and pancreatic duct dilatation between those two lesions. More PNEC showed well-defined margin than the PDAC (57.1% vs 25.0%, *p* = 0.04). Parenchymal atrophy was more common in PDAC than PNEC (67.9% vs 28.1%, *p* = 0.02). Positive lymph nodes or local invasion was more common in PDAC, but no significant differences were observed. The CT attenuation values (HU) of PNEC at arterial and portal phase were higher than those of PDAC (*p* < 0.05 or 0.01). Similar results were observed in contrast ratio (Fig. 2).

**Table 1:** Patient characteristics and CT imaging findings

Characteristics	PDAC(*n* = 28)	PNEC(*n* = 14)	*P* values
Age(years)	62.6 ± 9.7 (42–75)	56.4 ± 11.6 (25–71)	< 0.05
Gender			0.31
Male	17 (60.7%)	11 (78.6%)	
Female	11 (39.3%)	3 (21.4%)	
Size(cm)	3.56 ± 1.45	5.10 ± 4.42	0.09
Location			0.18
Head or neck	19 (67.9%)	6 (42.9%)	
Body or tail	9 (22.1%)	8 (57.1%)	
Margin			0.04
Well-defined	7 (25.0%)	8 (57.1%)	
Indistinct	21 (75.0%)	6 (42.9%)	
CT attenuation value(HU)			
Un-enhanced phase	33.8 ± 4.76	37.8 ± 5.86	0.23
Arterial phase	44.2 ± 8.56	64.6 ± 10.37	< 0.01
Portal phase	52.3 ± 7.49	64.9 ± 11.06	0.01
Parenchymal atrophy	19 (67.9%)	4 (28.6%)	0.02
Pancreatic duct dilatation	16 (71.4%)	7 (50.0%)	0.19
Positive lymph nodes or local invasion	14 (50.0%)	3 (21.4%)	0.10

*PDAC* pancreatic ductal adenocarcinomas; *PNEC* pancreatic neuroendocrine carcinoma

*CT* computed tomography

**Fig. 3:**
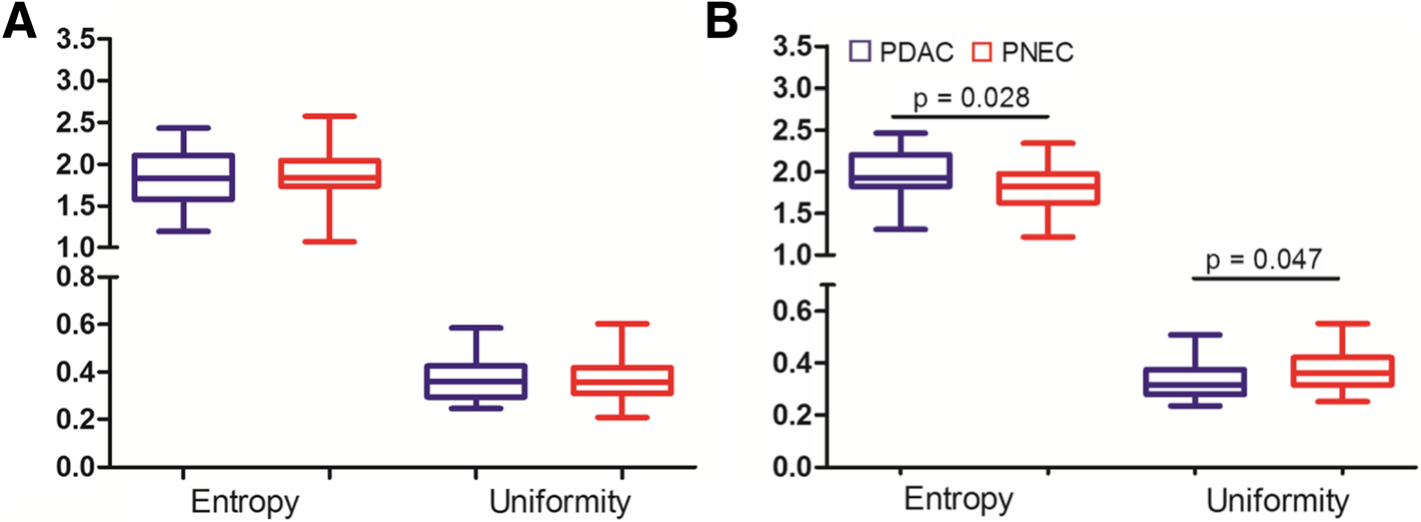
The entropy and uniformity in pancreatic neuroendocrine carcinoma (PNEC) and pancreatic ductal adenocarcinoma (PDAC) at the arterial (**a**) and portal (**b**) phases. PNEC showed lower entropy and higher uniformity than PDAC at the portal phase

Next, we examined the CT texture in PDAC and PNEC. The Kappa value for ROIs was 0.82. No significant differences were observed in kurtosis and skewness between PNEC and PDAC. Compared to PDAC at the portal phase, PNEC had lower entropy and higher uniformity (*p* < 0.05) (Fig. 3). However, no differences were observed at the arterial phase.

**Fig. 4:**
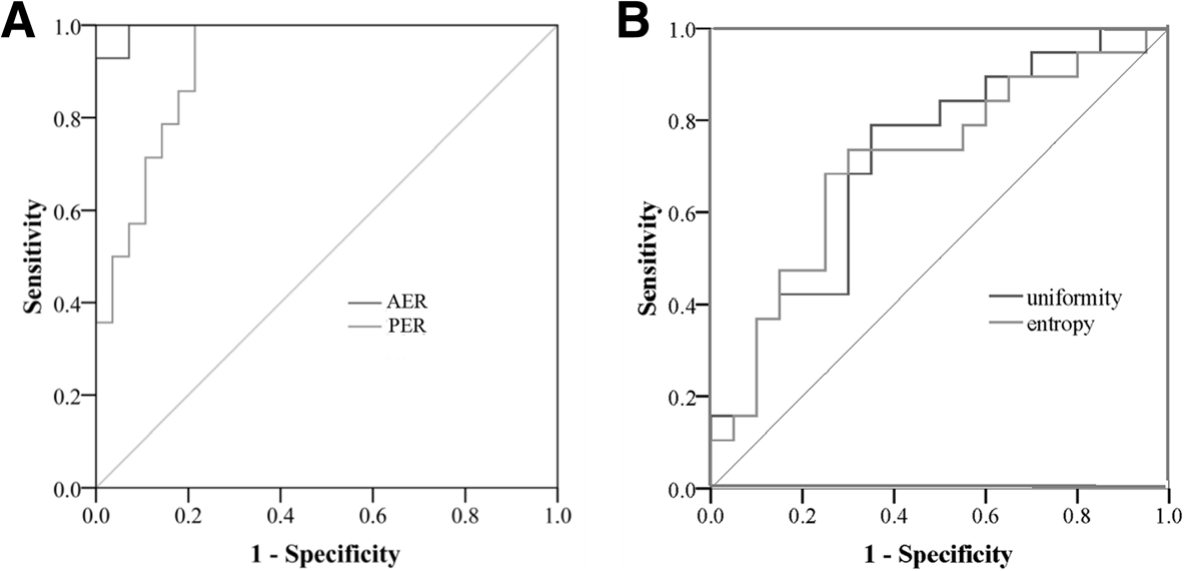
Receiver operating characteristic curves of the contrast ratio at the arterial phase (AER) and portal phase (PER) (A), and texture parameters (uniformity, uni; entropy, ent) (B) at portal phase for differentiating pancreatic neuroendocrine carcinoma (PNEC) from pancreatic ductal adenocarcinoma (PDAC). Entropy and uniformity at high sigma values had acceptable AUCs (> 0.70)

The sensitivity and specificity of the different imaging features for differentiating PNEC from PDAC ranged from 0.47–1.00 and 0.57–1.00 (Table 2, Fig. 4). AER and PER showed the higher AUC compared with other markers. For other imaging features, the AUC were 0.66–0.70. The sensitivity and specificity of the texture features (entropy and uniformity) for PNEC identification (vs. PDAC) ranged from 0.74–0.79 and 0.65–0.70 at the portal phase. The AUC were 0.71–0.72 at portal phase.

**Table 2:** Diagnostic performance of CT features and texture features for differentiating PNEC from PDAC

	Variables	AUC	Sensitivity (95% CI)	Specificity (95% CI)	Cutoff point
CT features	AER	0.99	1.0 (0.77–1.0)	0.93 (0.66–1.00)	0.56
	PER Size Margins Parenchymal atrophy	0.98 0.67 0.66 0.70	0.93 (0.66–1.0) 0.47 (0.28–0.69) 0.75 (0.55–0.89) 0.68 (0.48–0.84)	1.00 (0.77–1.00) 1.00 (0.72–1.00) 0.57 (0.29–0.82) 0.71 (0.42–0.92)	0.63 2.73
Texture features at portal phase	F3 uniformity	0.72	0.79 (0.54–0.94)	0.65 (0.41–0.85)	0.34
	F3 entropy	0.71	0.74 (0.49–0.91)	0.70 (0.46–0.88)	1.89

f1-f3 denote sigma values of 0.5, 1.5 and 2.5, respectively. *CI* confidence interval; *AER* enhancement ratio at arterial phase; *PER* enhancement ratio at portal phase; *AUC* area under the curve

## Discussion

PNEC is a rare pancreatic neuroendocrine neoplasm that is often misdiagnosed as PDAC on qualitative imaging. In the present study, we showed that quantitative imaging analysis, such as contrast ratio at arterial phase and portal phases, can differentiate PNEC from PDAC with good sensitivity and specificity. In addition, our data indicate that texture features (including entropy and uniformity) can also assist in differentiating PNEC and PDAC.

PNEC and PDAC have similar imaging findings, including hypovascular pattern on contrast-enhanced imaging and local or distal metastases. Despite this fact, only a few studies have examined the differentiation between hypovascular PNENs and PDAC. Jeon et al. [12] indicated that the MR enhancement pattern at portal phase or delayed phase was useful in differentiating between hypovascular PNENs from PDAC. They also showed that well-defined margin and lower frequencies of ductal dilatation were more common in hypovascular PNENs than PDAC. In the present study, we also showed that similar enhancement pattern in the portal phase (PER), and tumor margins were helpful in differentiating PNEC from PDAC. However, no differences were observed in ductal dilatation. PNETs G1/G2 with hypovascular enhancement may have been included in their study. In our current study, we only included PNEC G3. However, we previously found that contrast ratio at arterial phase and portal phase in MRI can potentially differentiate the two tumors [6]. Our data based on CT imaging are consistent with the previous findings. Those results demonstrated that quantitative imaging analysis is useful in differentiating PNEC and PDAC.

Interestingly, studies are finding that imaging texture analysis has great potential to improve cancer detection, staging, treatment and prognosis evaluation [13]. Several studies have demonstrated that texture features are valuable to grade PNENs [14–17]. However, the value of texture analysis in differentiating PNEC and PDAC is not well-understood. In our study, we found that PNEC had higher uniformity and lower entropy compared with PDAC on contrast-enhance images at portal phase. Entropy is a measure of randomness in the intensity of images. Entropy and uniformity reflect texture complexity and homogeneity in the tumors, respectively. Entropy is valuable in distinguishing malignant tumors from benign lesions [18]. Shindo et al. [19] showed that the entropy of ADC values in PDAC was higher than PNETs, which was consistent with our findings. Abundant fibrous stroma is typical histopathological features of PDAC. PNEC usually present more cellularity and a lower fibrous stroma [12]. Therefore, the complexity of enhancement in PDAC may be higher than that in PNEC. Consequently, low uniformity and high entropy are observed in PDAC. Although the ROC analysis showed that the diagnostic performance of texture parameters in differentiating PNEC from PDAC were not better than traditional quantitative indexes (i.e., AER and PER), texture analysis may be an important supplementary analysis for radiologists.

Our study has several following limitations. First, the sample size for PNEC is small due to the rarity of PNEC. Second, selection bias is unavoidable because our study is a retrospective study with single institution design. Third, scan parameters (e.g., slice thickness and reconstruction algorithm) may affect the texture analysis. Finally, since this was an exploratory study, only a few texture parameters were analyzed.

## Conclusions

Our data show that CT imaging features, including tumor margin, parenchymal atrophy, and contrast ratio at arterial phase and portal phase, are valuable in differentiating PNEC from PDAC. In addition, our data also indicate that assessing texture parameters – including entropy and uniformity –is a promising future direction for improving differentiation.

## Abbreviations

(AER: Contrast ratio at arterial phase
AUC: The area under the curve
CT: Computed tomography
HU: Hounsfield Unit
PDAC: Pancreatic ductal adenocarcinoma
PER: Contrast ratio at portal venous phase
PNEC: Neuroendocrine carcinoma
ROC: Receiver operating characteristics
ROI: Regions of interest
